# Development of a novel and viable knock-in factor V deficiency murine model: Utility for an ultra-rare disease

**DOI:** 10.1371/journal.pone.0321864

**Published:** 2025-06-02

**Authors:** Juan A. De Pablo-Moreno, Leopoldo González-Brusi, Andrea Miguel-Batuecas, Pablo Bermejo-Álvarez, Luis Revuelta, Antonio Liras

**Affiliations:** 1 Department of Genetic, Physiology and Microbiology, Biology School, Complutense University of Madrid, Madrid, Spain; 2 Animal Reproduction Department, INIA, CSIC, Madrid, Spain; 3 Department of Physiology, School of Veterinary Medicine, Complutense University of Madrid, Madrid, Spain; Versiti Blood Research Institute, UNITED STATES OF AMERICA

## Abstract

Factor V deficiency is a congenital coagulation disorder characterized by the absence or malfunction of factor V (FV). The purpose of this study was to develop a viable FV-deficient mouse model using CRISPR/Cas9 technology. A viable pathological model of the disease was not available to develop new therapies. A previous *in silico* study was performed to select a mutation causing a mild disease phenotype in humans (Thr1898Met missense). Such mutation was replicated in mice by CRISPR-mediated homology directed repair. Following crossing, homozygous individuals were subjected to coagulometry assays, including FV levels, prothrombin time (PT), and activated partial thromboplastin time (aPTT). The *in silico* study suggested that the mutation destabilizes FV structure of both mouse and human variants, putatively producing a mild phenotype of the disease in mice. Mendelian inheritance was observed in the offspring. No spontaneous signs of blood clotting disturbances, premature deaths or gestational dysfunctions were observed. FV levels in homozygous animals were 24.5% ± 5.1; 39.7 sec ± 2.8; PT was 61.8% ± 6.3; 23.4 sec ± 1.6 (INR = 1.47 ± 0.12); and aPTT was 46.9 sec ± 3.2. A viable FV-deficient mouse model was generated by introducing a missense mutation in FV. The model exhibits a mild phenotype of the disease, akin to that observed in humans.

## Introduction

FV deficiency is a recessive autosomal condition characterized by the presence of mutations in the *F5* gene [1]. 250 mutations have been referenced so to date in the *F5* gene, which are responsible for the disease [2]. Homozygous FV deficiency is regarded as an ultra-rare disease as it only occurs in 1–9 cases per million live births [1]. This disease is characterized by spontaneous hemorrhagic symptoms or exacerbated bleeds caused by trauma or surgical procedures. The most severe bleeds occur in internal organs such as the digestive tract or the central nervous system [3,4]. Severity of the disease is typically classified according to the levels of FV circulating in plasma. Thus, the disease phenotype is considered mild when FV levels are >10%, moderate when levels are between 1 and 10%, and severe when they are below 1% or undetectable. Clinical findings in patients with a mild phenotype include gingival bleeds or profuse bleeding after a surgical procedure [1,5–7].

Current treatment of FV deficiency is based on the administration of virally inactivated fresh-frozen plasma (FFP) [8,9] or platelet concentrates [10], as plasma-derived or recombinant FV concentrates are not commercially available. The implementation of advanced therapies such as gene or cell therapy, could constitute a therapeutic solution for this and many other hereditary conditions [11], and lay the foundations for the development of recombinant factors capable of treating the disease [12]. Against this background, coagulation factor complexes are an encouraging alternative. One such product is Octaplas^®^ [13], which allows optimal dosing as it contains standardized concentrations of different coagulation factors.

Animal models are highly valuable tools for generating biomedical knowledge, gaining a better understanding of the etiopathogenesis of various diseases, and developing therapeutic protocols based on biological, biotechnological and advanced therapy-based medications [9,14,15]. Mouse [16] and rat [17] models, together with dog [18] models, have been the most widely used animal models employed for the study of coagulopathies, with dog models being specifically used for hemophilia A and B.

As far as FV deficiency is concerned, the animal models employed to date have been based on knock-out (KO) mice or on zebrafish, the former generated through homologous recombination [19] and the latter through CRISPR/Cas9 technology [20]. FV KO mice are not viable, as the ablation often leads to developmental arrest, embryo resorptions, a reduction in full-term deliveries, and premature neonate death at best [19,20]. In addition, LMAN1-deficient mouse models have been described, which present a combined deficiency of FV and factor VIII (FVIII) [21,22]. In mice, FV activity is higher than that found in humans [23,24]. As in other species, FV is activated by the cleavage from inactive FV from its B domain, the protein’s phylogenetically least conserved region [1]. FV, which has both coagulant and anticoagulant properties, binds to activated factor X (FXa) following its activation by thrombin to transform prothrombin into thrombin [25–27]. At the same time, FV is inactivated by the interaction of activated protein C (APC) with protein S, giving rise to a trimeric complex [27,28].

The current lack of pathological models slows the pace of pharmacological research, both with respect to biological and biotechnological drugs, and to drugs based on advanced therapies. For other coagulopathies such as hemophilia A or B, pathological mouse models are already available, but for FV deficiency no viable pathological animal model has so far been developed. The current advances in genome editing through CRISPR technology currently allow to introduce point mutations at specific loci by direct zygote microinjection circumventing the generation of chimeric animals by genetically-modified ESC [29], which may serve to replicate point mutations causing human diseases.

The purpose of this study was to generate a viable FV-deficient mouse model for pharmacological research. Drawing on previous findings showing that very low FV concentrations result in non-viable animal models, a decision was made to generate a mild phenotype model, replicating a human mutation giving rise to a mild phenotype of the disease in a conserved region of mammalian FV. Phenotype characterization was performed by coagulometry assays to determine FV functionality, prothrombin time (PT) and activated partial thromboplastin time (aPTT).

## Materials and methods

### *In silico* study

An exhaustive study was conducted of the *F5* gene mutations contained in the Human Gene Mutation Database of the Institute of Medical Genetics in Cardiff (HGMD) [2] and in various scientific repositories such as PubMed^®^ [30]. Most mutations turned out to be missense mutations (change of one amino acid for another). A search was performed among these mutations to find homozygous mutations resulting in a mild FV deficiency characterized by factor levels above the minimum threshold. Subsequently, a multiple sequence alignment was performed of FV and FVIII proteins from different vertebrate species using the T-Coffee alignment tool [31], which combines different sequence alignment methods, to search for a mutation located in regions that were well conserved and were structurally and functionally distinctive. Eventually, a mutation was selected where the amino acid mutated in FV was found to be conserved in all the analyzed species. The mutation selected for its induction was identified as Thr1898Met (Thr1857Met in mice).

A similar search was carried out in the Wellcome Sanger Institute Database, the Mouse Genome Database and Ensembl to find out if the same mutation had been described previously in mice [32–34]. Following, the potential effect of the mutation in the mouse protein was modelled *in silico* by means of the Ensembl Variant Effect Predictor (VEP) (https://www.ensembl.org/Tools/VEP), which uses the “Sorting Tolerant From Intolerant” (SIFT) algorithm [34]. The algorithm, which assigns scores between 0 (deleterious) and 1 (tolerated), allows prediction of the impact on function due to mutation.

Next, an analysis was carried out to predict the stability of FV in its 3D structure. To do that, 3D models were generated of the native (inactive) and the activated wild type (WT) protein using the SWISS-MODEL homology modeling web server [35]. These models were run on the DUET server, applying the mCSM and SDM methodologies [36] to determine the impact of the chosen mutation on the stability of the protein’s structure. Pymol software (The PyMOL Molecular Graphics System, Version 2.5.5 Schrödinger, LLC) was used to visualize the structure of both the protein and the mutation. Lastly, online visualization of the 3D model was achieved by exporting a .gltf (GL Transmission Format) file of the protein to the Sketchfab repository (https://sketchfab.com), where it was stored and made available for visualization through the PyMol platform.

### Animals

In accordance with Royal Decree 53/2013 and EU Directive 2010/63/EU, the experimental study and the murine protocols were approved by Ethics Committee of the School of Veterinary Medicine of Complutense University of Madrid, the Ethics Committee for Animal Experimentation of the Complutense University, and the ethics committees of the National Institute for Agrarian Research and Technology (INIA) and the Madrid Regional Government (PROEX 358.4/21, PROEX 040/17). Mice (CBAXC57BL6 F1 hybrids) were maintained on a 12/12-hour light/dark cycle at optimal temperature, fed a standard diet (Altromin, Altromin International, Lage, Germany), allowed *ad libitum* water intake, and subjected to strict veterinary control. At the end of the experiment, mice were sacrificed using CO₂ exposure, with death confirmed by cervical dislocation, and all efforts were made to minimize suffering.

### sgRNA and ssDNA preparation

CRISPR-mediated homology directed repair (HDR) was employed to generate the single T-C substitution involved in Thr1857Met mutation (ACG to ATG codon methionine), following methods previously employed by the group [37]. The strategy was based on the use of single-guide RNA (sgRNA) targeted at the closest PAM to the intended base conversion site and a single-stranded DNA (ssDNA) donor with 50 bp homology arms, which contained the selected mutation as well as a conservative mutation in its PAM region to avoid CRISPR recognition following the editing process (Table 1). sgRNA was synthesized and purified using the Guide-it sgRNA In Vitro Transcription Kit^®^ (Takara Bio, Shiga, Japan); ssDNA and the oligonucleotides were acquired from Integrated DNA Technologies (Integrated DNA Technologies, Coralville, Iowa, USA), and the capped polyadenylated Cas9 mRNA was generated through in vitro transcription using the mMESSAGE mMACHINE T7 ULTRA kit^®^ (Life Technologies, Carlsbad, California, USA) with Bbsl (New England Biolabs, Ipswich, Massachusetts, USA)-linearized plasmid pMJ920 (Addgene 42234) serving as a target [38]. The mRNA was purified using the MEGAClear kit (Life Technologies, Carlsbad, California, USA).

**Table 1:** sgRNA and ssDNA constructs.

	Bp	Sequence (5′–3′)	Position and observations
sgRNA target	20	GCGTTTGCATGCCAGCTACC	*F5* gene exon 18: 5678–5698
Donor ssDNA	100	GCATCCAAGCCTGGCTGGTGGCTCCTA GACACAGAGGTTGGAGAAAATCAGGT AGCTGGCATGCAAATGCCATTTCTCATC ATAGACAAAG**GTATCACTT**^a^	*F5* gene exon 18: 5602–5718 C>T 5695 missense mutation C>T 5675 synonymous mutation

^a^The nucleotides in bold are the starting point of the intron 19 region of the *F5* gene; bp: size of the product expressed in base pairs.

### Microinjection

Superovulation was induced in 18 (7–8-week-old) hybrid donor female C57BL/6JOlaHsd mice by means of intraperitoneal injections of 5 IU of pregnant-mare serum gonadotropin (PMSG, Folligon, MSD Animal Health) and an equivalent dose of human chorionic gonadotropin (hCG, Veterin Corion^®^, Divasa-Farmavic) administered at 48-hour intervals. The induced females were mated with CBA/CaOlaHsd stud males and the zygotes were retrieved from the oviducts.

Microinjections of mouse zygotes were performed with a micromanipulation system (Eppendorf Transferman & Femptojet) equipped with a Leica DMi8 inverted microscope. An injection of 3–5 pl of an admixture of 100 ng/µl of Cas9-coding capped polyadenylated mRNA, 50 ng/µl of sgRNA and 10 ng/µl of donor ssDNA was administered to the cytoplasm of each zygote with a microinjection filament needle of approximately 1 µm in diameter, using positive pressure to transfer the solution from the needle to the cytoplasm (Eppendorf Transferman & Femptojet). After the microinjection, the embryos were cultured in EmbryoMax KSOM Mouse Embryo medium (Millipore) at 37°C in a water-saturated atmosphere (90% N_2_, 5% CO_2_ and 5% O_2_ ) for four days, until they reached the blastocyst stage [39]. Blastocysts were transferred to pseudo-pregnant Swiss recipients 2.5 days post-coitum (dpc) by the utero-tubal technique [40].

### Genotyping

Genomic DNA samples from ear biopsies from the surviving pups were prepared using a FavorPrepTM Tissue Genomic DNA Extraction Mini Kit (Favorgen, Vienna, Austria). Genotyping was performed by deep sequencing to detect all alleles harbored by each individual. The procedure consisted on a first PCR amplifying the region containing the target sequence using primers that contained Illumina overhangs, and a second PCR to introduce unique indexes suitable for Illumina sequencing (Table 2), as previously described protocols [38]. The first PCR was conducted under the following conditions: 95°C, 2 min; 25 x (94°C, 20 sec, 60°C, 30 sec, 72°C, 30 sec), with a 5-minute final elongation stage at 72°C and maintenance at 8°C. Table 2 provides details on the amplified fragment of the WT sequence. The products of the first PCR assays were purified using AMPPure XP beads (Beckman Coulter, Brea, California, USA) and libraries were prepared by an index PCR, which added Illumina adaptors and indexes identifying each pup using Nextera XT (Illumina, San Diego, California, USA). Libraries were purified, pooled to 4 nM and sequenced on the Illumina MiSeq platform providing 250 bp paired-end sequencing reads. Individual alleles were identified following a quality control stage and mapped to reference; variant calling was also performed.

**Table 2:** Genotyping primers.

	Construct (5′–3′)	Access number	WT bps
Forward	^a^*TCGTCGGCAGCGTCAGATGTGTATAAGAGACAG* AGAGAGCTCCGTCACAACAT	NC_000067.7	358
Reverse	*GTCTCGTGGGCTCGGAGATGTGTATAAGAGACAG* GCCCGTCTTCACATTTTCACA	NC_000067.7	358

^a^llumina MiSeq System sequencing overhangs are identified in italics within the genotyping primers; pb WT: size of the wild-type product expressed in base pairs.

A founder female carrying the mutation intended (Thr1857Met) and a KO allele formed by a 4 bp frame-disrupting indel was crossed with a WT male, and the heterozygous (Hz) F1 individuals carrying the Thr1857Met mutation were intercrossed to obtain WT, Hz and homozygous (Hm) individuals with the Thr1857Met mutation. The subsequent genotyping was carried out by subjecting the PCR products to Sanger sequencing.

### Blood collection

Six WT, six Hz and six Hm animals were selected in a 1:1 male/female ratio from each group. To obtain blood samples, animals were manually restrained without anesthesia. Blood was extracted during the first six hours of light by means of a puncture in the submandibular venous plexus with a 23G needle. A total of 0.25 ml of blood was obtained, which was collected in sodium citrate tubes containing 3.2% citrate (0.109 M) (Vacutest Kima, Arzegrande, Padua, Italy). To plot the standard curves, blood was extracted from the WT mice to obtain plasma pools. The samples were centrifuged at 2,500 g for 15 min at 20°C and then aliquoted in Eppendorf tubes for immediate coagulometric measurement.

### Hematological and coagulation tests

Determination of FV levels, PT and aPTT was carried out in a STart Max II R (Diagnostica Stago S.A.S. Barcelona, Spain) viscosity-based coagulometer in accordance with the manufacturer’s instructions. Measurements were made by placing a series of cuvettes in the coagulometer (Start 4 Cuve, Diagnostica Stago S.A.S. Barcelona, Spain) to which metal beads were subsequently added (Diagnostica Stago S.A.S. Barcelona, Spain). All reagents were prepared according to the manufacturer’s guidelines. Once all the reagents and the sample had been pipetted into the cuvettes, the reaction was initiated. The process was automatically completed once clot formation occurred. Concomitantly to these determinations, positive and negative controls were prepared from the System Control N/P reagents (Diagnostica Stago S.A.S. Barcelona, Spain). Calibration curves were drawn using plasma from WT mice. Dilutions and reagents were employed in the measurement process following the protocol described by De Pablo-Moreno et al. [23]. 1:100 and 1:3 dilutions were used, respectively, for measuring FV levels and PT. The standard curves for FV levels and PT can be found in the supplementary materials section. Both FV levels and PT were expressed in seconds and percentages (%). The INR was also calculated as a ratio of the PT of each sample raised to the power of the ISI value of the neoplastin used. aPTT was measured in a 1:1 dilution, with results expressed in seconds.

### Statistical analysis

The statistical analysis was carried out with the SPSS 27, v9.4 software package (SAS Institute, Cary, NC, USA). The FV and PT calibration curves were generated using Excel (Microsoft Office 365) and all the graphs were designed with GraphPad Prism 8 software (GraphPad Software, La Jolla, CA, USA). The Shapiro-Wilk normality test was used for all the tested variables. The Levene test was conducted to determine the variance of homogeneity across the groups. The differences between the experimental groups and the control group (WT), as well as the differences between the Hz and Hm groups were evaluated using Student’s t-test to calculate differences between means. Statistical significance was set at a p-value <0.05.

## Results

### *In silico* study

The review carried out of over 250 mutations in the human *F5* gene revealed a considerable number of missense mutations. A subsequent analysis made it possible to identify mutations in human homozygous patients where FV levels were above 10% (mild FV deficiency). Moreover, following multiple sequence alignment, the mutated amino acid in the selected mutation was found to be conserved and located in highly conserved regions of all the analyzed species. Of all the resulting mutations, Thr1898Met (ACG-ATG) was the mildest surpassing the minimum threshold for mild FV deficiency in homozygosity. This mutation, first described by Délev et al. [41], was present in a male individual with FV levels of 26% (measured by a chromogenic assay) and no hemorrhagic symptoms, who carried the mutation in homozygosity. The mutation, located in exon 18, which corresponds to A3 domain of FV, was also found in a compound heterozygous male patient who also presented a mild phenotype of the disease (FV levels, 10%) but who developed symptoms such as hematomas and epistaxis. This patient also presented the Gly926fsX4 mutation.

**Fig 1:**
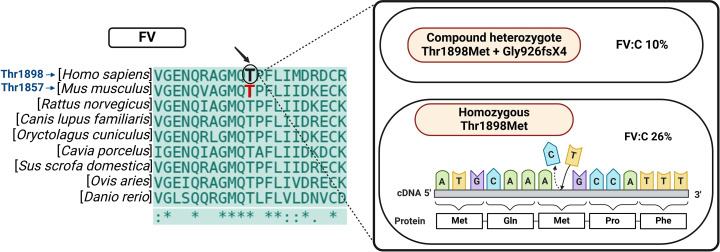
Multiple Sequence Alignment of factor V (FV) across species. Multiple alignment of FV sequences, corresponding to Thr1898 region in *Homo sapiens*, *Mus musculus*, *Rattus norvegicus*, *Canis lupus familiaris*, *Oryctolagus cuniculus*, *Cavia porcelus*, *Sus scrofa domestica*, *Ovis aries* and *Danio rerio*. The mutated amino acid can be seen at the center; the homologous amino acid in mice corresponds to Thr1857 in FV (in red). Abbreviations: F, factor; C, chromogenic.

An analysis of the area where the mutation occurred in various vertebrate species showed a high degree of conservation of FV (Fig 1). FVIII also showed strong conservation in the homologous region, as presented in S2 Fig. Sequence alignment of both proteins revealed that the site occupied by Thr1898 in humans is occupied by Thr1857 in mice.

Several missense mutations have been described in the literature, particularly within the realm of FVIII. Lombardi et al. [42] reported a homozygous mutation in amino acid 2031 (Thr2031Ile), homologous to Thr1898 in FV, with FVIII:Ag levels of 27.6% ± 5.1 and FVIII:Ac levels of 20.6% ± 3.2. The mutation was described as mild in a male homozygous patient. It was also observed that the adjacent area also harbored several previously described mutations in FVIII (S2 Fig). Concerning FV, no mutations have been reported in the literature in the adjacent area.

After analyzing inter-species phylogenetic conservation in FV and FVIII and confirming that the site where the mutation occurred in the mouse corresponded to Thr1857, a search was conducted in several repositories to find out whether the human Thr1857Met mutation had been described previously in a mouse model. The search did not return any results.

The SIFT algorithm was run on Ensembl Variant Effect Predictor software (VEP) to predict the potential impact of the missense mutation on the mice. The mutation was identified as ENSMUST00000086040.6:c.5570C>T. The software predicted that the mutation would exert a moderate impact. The SIFT algorithm assigned the mutation a score of 0, which corresponds to a deleterious mutation.

To determine how the stability of the murine FV protein might be affected by the selected missense mutation, a 3D prediction of the mutation in the inactive FV protein was generated using the SWISS MODEL predictor. This process produced a *.pdb* file with a 3D predictive model. This 3D model for the inactive FV (S3 Fig) was mapped on a Ramachandran plot, which revealed that 75.06% of amino acids were within favored regions with a MolProbity Score of only 2.05.

The MolProbity score was low due to the presence of amino acids pertaining to the B domain, which is cleaved once the FV protein becomes activated. To compare the structure of FV in its active and inactive forms, a 3D model for activated FV (FVa) was generated using the SWISS MODEL predictor, which yielded a score of 93.36% on the Ramachandran plot and a MolProbity score of 1.16.

Both 3D models were run on the mCSM, SDM and DUET (ΔΔG) servers to predict the stability of the FV protein in a mouse model in the presence of mutations Thr1857Met for inactive FV and Thr1031Met for activated FV. The analysis showed that the mutations gave rise to a less stable protein variant in both FV forms. The stability of inactive FV was recorded at -0.083 kcal/mol, -0.53 kcal/mol and -0.124 kcal/mol, respectively, on each server, while the stability of activated FV was recorded at -0.072 kcal/mol, -0.43 kcal/mol and -0.114 kcal/mol respectively.

In addition, a representation of both proteins and the mutated amino acid were generated in Pymol. Both proteins were included in the Sketchfab open access repository in *.gltf* format where they can be visualized in 3D.

### Generation of a FV-deficient mouse model

**Fig 2:**
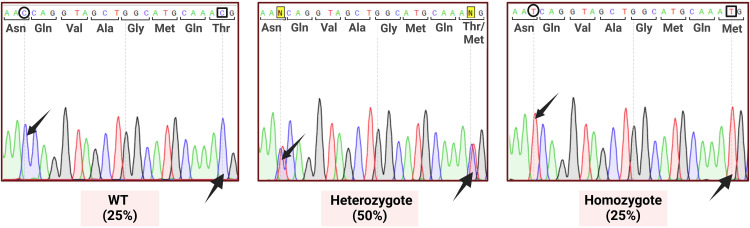
Sanger sequencing chromatograms confirming the Thr1857Met mutation in mice. The figure shows the sequencing chromatograms of mice carrying the mutation as well as the chromatograms for WT, heterozygous and homozygous individuals. Abbreviations: WT, wild type.

Making use of ssDNA-mediated HDR repair and CRISPR/Cas9 technology, a knock-in model was created by microinjecting zygotes in a mixed CBAXC57BL6 F1 background to induce the Thr1898Met mutation, which in humans produces a mild disease phenotype with FV levels of 26% and without spontaneous bleeds. The genome editing approach employed comprises two steps: first, the CRISPR system induces a DSB close to the base pair to be modified and, secondly, HDR repairs the DSB using the ssDNA donor provided, which contains the intended mutation. Specifically, the ssDNA carried the missense Thr1857Met (C>T 5695) mutation in the mice as well as a conservative mutation (C>T 5675) in the PAM to prevent target recognition once the mutation had been introduced. The latter mutation was conservative, converting codon AAC to AAT, both encoding for asparagine. The drawback of employing the CRISPR system to introduce point mutations is that the DSB can be generated by non-homologous end joining (NHEJ) instead of HDR. NHEJ typically repairs the DSB, introducing random mutations (indels) that may disrupt *F5* functionality leading to non-viable embryos. This situation probably occurred in our case, as survival to term (i.e., pups delivered/embryos transferred) was substantially lower (14/70, 20%) than usual (~70% for non-manipulated animals) [40]. Genotyping revealed that most (12/14) pups were edited, 4 were mosaic (contained more than 2 alleles) and one (1/14) contained the intended mutation. The sequences of all alleles of the 12 edited pups can be found in S4 Table. The pup containing the intended mutation was a heterozygous female carrying the intended allele and a 4 bp deletion. Such a female was chosen as founder and crossed with a WT male to obtain an F1 of Hz individuals carrying the intended mutation and a WT allele. This Hz F1 was subsequently intercrossed to obtain the WT, Hz and Hm animals required for a coagulometric study. The crosses performed followed the expected Mendelian proportions indicating that Hm individuals were viable (Fig 2).

### Clinical findings

No spontaneous bleeds were observed in any of the homozygous individuals. During differential ear tagging, WT and Hz animals did not experience any hemorrhage while Hm individuals did exhibit short-duration bleeds. Moreover, F1 intercross (Hz x Hz) resulted in normal litter sizes following Mendelian proportions, fetuses developed to full term and no spontaneous deaths were observed for the length of the experiment.

### Coagulometric analyses

#### FV levels.

**Fig 3:**
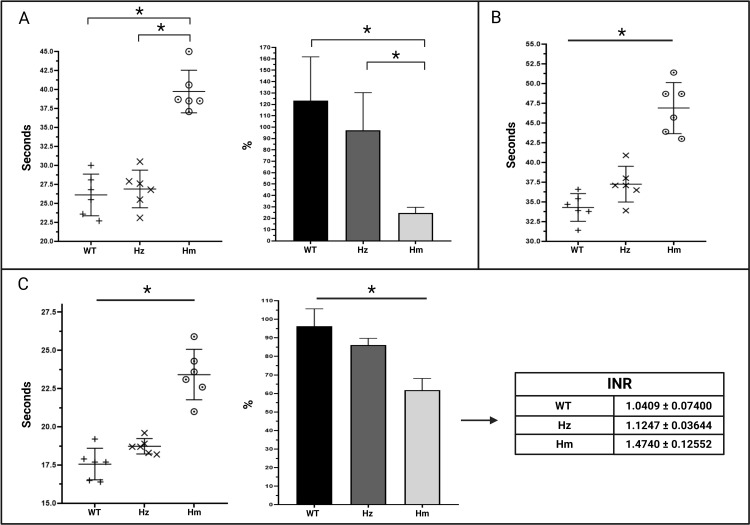
Coagulometric measurements. A) FV level measurements of the WT, Hz and Hm groups expressed in seconds and percentages (n=6). B) aPTT measurements of the WT, Hz and Hm groups expressed in seconds (n=6). C) PT measurements of the WT, Hz and Hm groups expressed in seconds, percentages and INR (n=6). The data is expressed as mean ± standard deviation. *p < 0.05 according to Student’s t-test. Abbreviations: FV, factor V; WT, wild type; Hz, heterozygote; Hm, homozygote; PT, prothrombin time; INR, International Normalized Ratio; aPTT, activated partial thromboplastin time.

The values obtained (Fig 3) were expressed as time-to-clot-formation (in seconds) and as percentages over a standard curve generated based on a pool of plasma from WT individuals. Mean FV levels were 26.1 sec ± 2.8 (123.2% ± 38.5) in WT individuals; 26.9 sec ± 2.5 (97.2% ± 33.0) in Hz individuals; and 39.7 sec ± 2.8 (24.5% ± 5.1) in Hm individuals. Normal distribution and equal variances were observed between the groups. Student’s t-test found statistically significant differences between the WT and the Hm group (p<0.001) and between the Hz and Hm groups (p<0.001) (Fig 3A).

#### aPTT.

The values obtained were expressed as time-to-clot-formation (sec). Mean aPTT was 34.3 sec ± 1.8 in WT individuals; 37.2 sec ± 2.3 in Hz individuals; and 46.9 sec ± 3.2 in Hm individuals. Normal distribution and equal variances were observed between the groups. Student’s t-test indicates statistical significance between the WT and the Hz group (p=0.031), between the WT and the Hm group (p<0.001) and between the Hz and the Hm group (p<0.001). The data is shown in Fig 3B.

#### PT.

The values obtained were expressed as time-to-clot-formation (sec) and as percentages over the standard curve generated based on a pool of plasma from WT individuals. Mean PT was 17. 6 sec ± 1.0 (96.3% ± 9.3) in WT individuals; 18.7 sec ± 0.5 (86.0% ± 3.6) in Hz individuals, and 23.4 sec ± 1.6 (61.8% ± 6.3) in Hm individuals. Normal distribution and equal variances were observed between groups. Student’s t-test revealed statistically significant differences between the WT and Hz groups (p=0.032), between the WT and the Hm group (p<0.001) and between the Hz and the Hm group (p<0.001). The data is shown in Fig 3C. The INR was also calculated for each group based on the plasma of a normal WT individual, with a PT of 17 sec and an ISI of 1.21. The INR was 1.04 ± 0.07 for WT individuals, 1.12 ± 0.03 for Hz individuals, and 1.47 ± 0.12 for Hm individuals.

## Discussion

The present study set about generating a FV-deficient mouse (*Mus musculus*) model by introducing a missense mutation capable of producing a mild-phenotype FV deficiency in humans. *In silico* studies cannot provide hard-and-fast predictions determining phenotypic realities —because other factors such as the physiologic microenvironment do play an important role in dictating the phenotypic effect of a given mutation— but they have been useful for a preliminary prediction in a mouse model. *In silico* studies make it possible to predict the effects of a mutation in an *in vivo* model [43,44], minimizing the number of experimental animals required to comply with the refinement requirement of Russell & Burch’s Three Rs [45].

The generation of the model required to introduce a single point mutation, which was accomplished by taking advantage of the enhanced efficiency of combining HDR with the CRISPR system. Genomic insertion by HDR alone (i.e., by solely providing a recombination template) is a very inefficient technique (0.1%) [46], as it relies on the homonymous DNA repair mechanism to introduce the mutation, such endogenous mechanism remaining inactive when the target sequence is intact. However, when combined with CRISPR, the DSB generated by the system at the target sequence activates HDR, boosting the efficiency of the targeted DNA insertion [29]. To prevent off-target effects, we delivered the system as RNA instead of plasmid given that RNA injection results in transient activation of the CRISPR system, which minimizes the odds for off-target mutations as evidenced by prior models generated by our group in diverse mammalian species where no off-target mutations were observed [38,39,47,48].

The timing of CRISPR delivery (late zygote stage) is prone to generate mosaic embryos, as genome modification will probably occur after DNA replication [49], before the mutation is generated, leading to the occurrence of more than two mutation events (i.e., the presence of more than two alleles and thereby more than one cell type) in the embryo [49]. This situation is undesirable when attempting direct generation of KO animals, but when a base substitution is intended, mosaicism increases the odds of obtaining a founder carrying at least one allele with the intended mutation. The higher the number of alleles carried by an individual, the more probable it would be that at least one of them would be the intended. This rationale has led to a deliberate search for mosaicism when aiming targeted DNA insertions by delivering CRISPR components at the two-cell stage [50]. However, despite the probable presence of mosaicism at preimplantation stages of development following late zygote injections, only 4/12 edited pups (33%) were mosaic, which suggest that lineage segregation corrected mosaicism by ultimately forming the epiblast (the lineage forming the embryo proper) from a single cell type.

The model, by CRISPR-mediated HDR, constitutes the only available viable *F5*-mutant vertebrate model, as prior models caused prenatal or neonatal death. This model represents the mild phenotype of the human disease and could become a useful tool for testing new biological, biotechnological and gene or cell therapy-based drugs for the treatment of FV deficiency. The Thr1898Met mutation described in humans is a missense mutation affecting the functionality and structural integrity of the FV protein [41], similar to other missense mutations responsible for other clotting disorders such as hemophilia A and hemophilia B [51,52]. Missense mutations account for over 50% of the mutations identified in genetically-determined coagulation disorders [53] and typically result in mild disease phenotypes. In the case of FV deficiency, they usually induce FV levels >10%, with the disease sometimes remaining undiagnosed because of a lack of symptoms [7,54,55], which is also observed in other coagulopathies [51,52,56–58]. In addition, missense mutations have played an important role in other animal models for example those related to hemophilia B [59].

As far as phylogenetic studies are concerned, they are useful not only to determine the state of conservation of amino acids and nucleotide sequences but also to identify homologous regions in other animal species. This makes it possible to locate structurally or functionally well-preserved areas in various species [60]. Phylogenetic studies have also allowed a predictive approach to the impact on function of mutations that result in an amino acid change [61].

The region of the FV protein subject to analysis, i.e., the area where the mutation occurred exhibited a high degree of interspecies conservation, which may be due to the fact that was located in the A3 domain, one of the best conserved domains in FV according to the literature [62,63]. This high degree of conservation is not only found among mammal species, but also in the zebrafish, one of the teleost species presenting a large number of orthologous genes associated with homeostasis [64]. These findings could be interpreted as evidence for the functional importance of the region where the mutation occurred.

The similarity between the FV and FVIII proteins is not limited to their structures but also extends to their functional activation and inactivation process, the former mediated by thrombin and the cleavage of the B domain and the latter caused by obliteration of the A2 domain by APC [65]. FV shares approximately 40% sequence homology with FVIII, with its B domain being its least conserved domain [66]. For that reason, it is important for any study on mutations to verify the homology between both proteins [41,67]. The present analysis found a high level of conservation across species between the regions of FVIII and FV sharing the greatest homology. Moreover, a mutation in homologous amino acid has been reported to result in a mild disease phenotype [42]. All of this would seem to indicate that the Thr1898 amino acid could play a leading role in the functionality of FV.

Concerning 3D modeling on the Swiss Model server, the reliability of the model can be measured using the Ramachandran plot [35]. A model must be generated of both inactivated and activated FV as the inactive protein harbors a larger number of amino acids in unfavored regions such as B domain of the protein, which is cleaved on activation [27]. Algorithms such as SIFT, which predicts potential functional consequences, and mCSM, SDM and DUET, whose predictions are related to the stability of the protein, have been shown to be highly reliable [36,68]. In our case, the values obtained correspond to a deleterious mutation that results in a negative energy balance when one amino acid is replaced by the other. In the case of the Thr1857Met variant (Thr1034Met for FVa), exposure of the polar residue (threonine) rather than the non-polar one (methionine) on the surface could jeopardize the stability of the protein. Similarly, the substitution of a hydrophobic residue for a hydrophilic one prevents water molecules from gaining access to the protein, which destabilizes its structure [69].

Previous studies have described knock-out animal models where severe FV deficiency developed following truncation of the protein. However, none of the animals in these models were able to survive [19,20]. One of the limitations associated with the FV-deficiency models proposed so far has been that they provoked alterations in the development of the vasculature and the yolk sack during embryogenesis. According to several authors, such alterations result from the inability of thrombin to bind to PAR receptors. The absence of FV production in these knock-out models entails a failure in prothrombin activation which, in turn, leads to a severe disruption of embryogenesis in the form of a resorption of embryos during pregnancy, the inability of embryos to survive to term or premature neonate death in the best-case scenario [19,20,70].

The litter obtained from the cross of Hz individuals were of normal size and followed Mendelian proportions compatible with no effect on pre-term or neonatal viability of the mutation, which contrast to the effects of more severe mutations on FV [71]. In contrast to severe mutations truncating the protein and thereby dramatically reducing protein activity, missense mutations do not block completely protein activity. This means that the procedure results in a viable animal model of FV deficiency that could be used as a basis for preclinical studies. Generation of this viable mouse model was possible thanks to the CRISPR/Cas9 and HDR systems, which have proven highly effective for generating pathological animal models [72–74].

Clotting rate is significantly higher in mice than in humans [23,24,75] and thereby it was necessary to generate a specific protocol to measure FV levels in mice [23,76], to reduce the amount of blood extracted in order to minimize animal discomfort, and to optimize the overall effectiveness of the technique [77]. A previous study by our group found normal FV levels in a mouse model (95.80% ± 18.14) [23]. The results of the coagulometric analyses conducted in the present study with respect to FV, PT and aPTT exhibited high clotting rates, in line with previously published data on WT individuals [23,24,76,78]. The valuation of FV levels, PT and aPTT showed a trend toward lower clotting rates in Hz individuals. These results are in accordance with those obtained in heterozygous human patients who present with longer clotting times, with significant fluctuations across individuals [79,80].

Although Hm human individuals have been shown to exhibit lower FV levels than WT individuals, the reduction observed in knock-out mouse models —characterized by missense mutations and a mild phenotype— was too severe (FV levels below 0.01%) [7,19,54,55,71]. However, it must be considered that, in human patients with FV deficiency, unlike in those with hemophilia A or B, deficient factor levels are not related to the presence of clinical symptoms [7]. Indeed, higher factor levels could result in fewer symptoms and a milder course of the disease [1].

A comparison with hemophilic mice is not possible with respect to PT as the FVIII route corresponds to the intrinsic pathway of the coagulation cascade (aPPT) [81]. A study that generated FX-deficient mice [82] obtained longer PT values for those animals as compared with WT individuals. However, these results are not comparable with the findings of this study given that the methodology used is different and that the authors did not use the INR. The results of a previous study by our group using the methodology described here showed mean PT values of 104.31% ± 14.52 in healthy mice. In humans, PT values are typically normal in individuals with PT levels above 70% [83]. In our study, shortening PT was not critical as the mutation under analysis results in a mild phenotype, which means that a longer PT may be offset by the activity of other coagulation factors and by the activity of FV itself, as minimal as it may be. This finding is common in patients with mild FV deficiency, who tend to exhibit shorter clotting times, sometimes even within the normal range, than individuals with a severe phenotype [84,85]. The INR normalizes the PT value in a plasma control taking into consideration the ISI of the neoplastin used. The index is more commonly used in patients with thrombotic problems who are on anticoagulant therapy. Such patients typically have an INR between 2–4, which reduces and controls their thrombotic risk [81,86,87]. This parameter is also used in patients with coagulopathies, who usually have higher INRs [55,81]. This was precisely the case of the animal model generated in this study. INR could become a useful parameter for animal models as it would allow comparisons across different studies, measurement techniques and even species.

As regards aPTT in homozygous individuals, although our study did find longer aPTTs, the values were not as high as those found in mice with hemophilia A or B [16]. This difference is probably attributable to the mild phenotype of the disease in our mice [84,85]. In humans, aPTT typically ranges between 30 and 33 seconds, which is very close to the values obtained in our previous mouse study [23,88].

The present study, which constitutes the first achievement of obtaining a viable FV-deficient mouse model, has succeeded in generating the intended mutation —identified through next-generation sequencing— obtaining a mild phenotype of the disease as determined by the FV, PT and aPTT levels measured. Although the coagulometric assays carried out were successful in establishing the disease phenotype in the model obtained, which was the ultimate goal of this article, our group intends to conduct further research in the medium term including the generation of thrombin in the presence of thrombomodulin to analyze the protein C (PC) pathway; platelet tests; the evaluation of other coagulation factors and fibrin degradation products; thromboelastometry; routine blood tests; and antigen quantification assays.

## Conclusions

CRISPR-mediated HDR allows the generation of point missense mutations in animal models to emulate a human equivalent. The mutation induced in this study resulted in viable individuals with mild FV deficiency. In contrast to other congenital conditions such as hemophilia A and B, where animal models have been in use for a long time, a viable model has to date not been obtained for FV deficiency. The model described in this study could be employed to test new biological or biotechnological drugs as well as new gene and cell-therapies. Availability of a model such as the one presented here could facilitate collaboration between different research groups and, eventually, promote the advancement of preclinical pharmacological research into rare and ultra-rare conditions such as FV deficiency.

## Supporting information

S1 Fig Generation of the Thr1857Met mutation in the mice by means of CRISPR-mediated HDR. Abbreviations: F, filial generation; WT, wild type.
(TIFF)

S2 Fig Multiple sequence alignment of the homologous region of factor VIII (FVIII) across species. Multiple alignment of FVIII sequences, corresponding to Thr1898 homologous region in *Homo sapiens*, *Mus musculus*, *Rattus norvegicus*, *Canis lupus familiaris*, *Oryctolagus cuniculus*, *Cavia porcelus*, *Sus scrofa domestica*, *Ovis aries* and *Danio rerio*. The mutated amino acid can be seen at the center; the homologous amino acid in mice corresponds to Thr2031 in FVIII (in black). Abbreviations: F, factor; A, Antigen.
(TIFF)

S3 Fig 3D Models of Inactive and Activated factor V. A) Graphic representation of the structure of inactive FV as predicted by the SWISS MODEL tool and the Ramachandran plot. B) Graphic representation of the structure of activated FV as predicted by the SWISS MODEL tool and the Ramachandran plot. Pymol visualization of WT threonine (green) and the mutated amino acid methionine (red). The QR code in the figure provides access to the 3D representation of each protein in the Sketchfab repository. Direct links: inactive FV, https://n9.cl/fvinactive; activated FV, https://n9.cl/fvactive_.
(TIF)

S4 Table Sequences of the alleles found in edited pups. The WT allele is shown in the first raw for comparison. Edited alleles can be formed by base substitutions (marked in green letters), base deletions (marked by -) or base insertions (marked in red). The sought after allele is highlighted in blue).
(DOCX)

S5 Fig Calibration curves. A) Calibration curve for FV. B) Calibration curve for PT. Abbreviations: FV, factor V; PT, prothrombin time.
(TIF)

S6 Table Coagulometric measurements. Factor V measurements (expressed as % and seconds), prothrombin time measurements (expressed as %, seconds, and INR), and activated partial thromboplastin time measurements (expressed in seconds) for the WT, HZ (heterozygous), and HM (homozygous) groups. ^a^Percentage obtained from the standard curve based on the plasma WT *pool*. ^b^INR calculated as the ratio of the individual sample value to the normal sample value raised to the ISI.
(DOCX)
