# Supplementary figures and images for: Development of a novel and viable knock-in factor V deficiency murine model: Utility for an ultra-rare disease
Source: PLoS One. 2025 Jun 2;20(6):e0321864. doi: 10.1371/journal.pone.0321864 (PMC12129228; doi:10.1371/journal.pone.0321864)

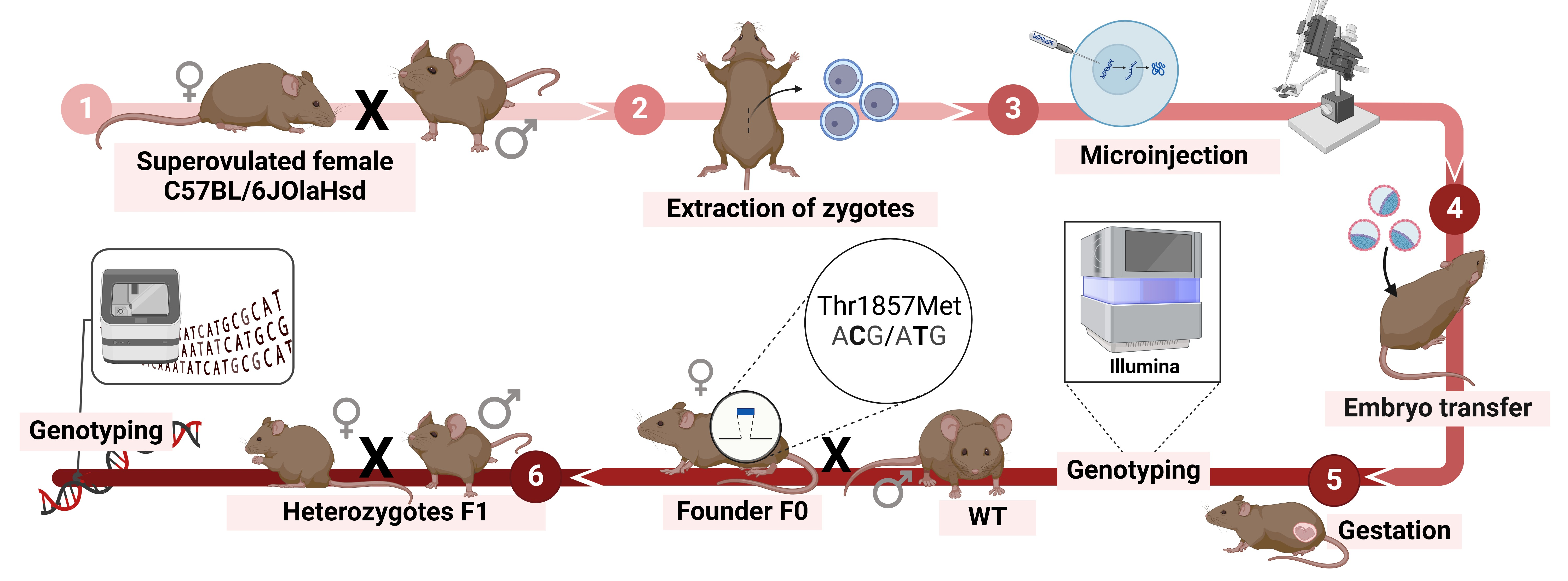

Supplement: S1 Fig — Abbreviations: F, filial generation; WT, wild type. (TIFF) [file pone.0321864.s001.tiff]

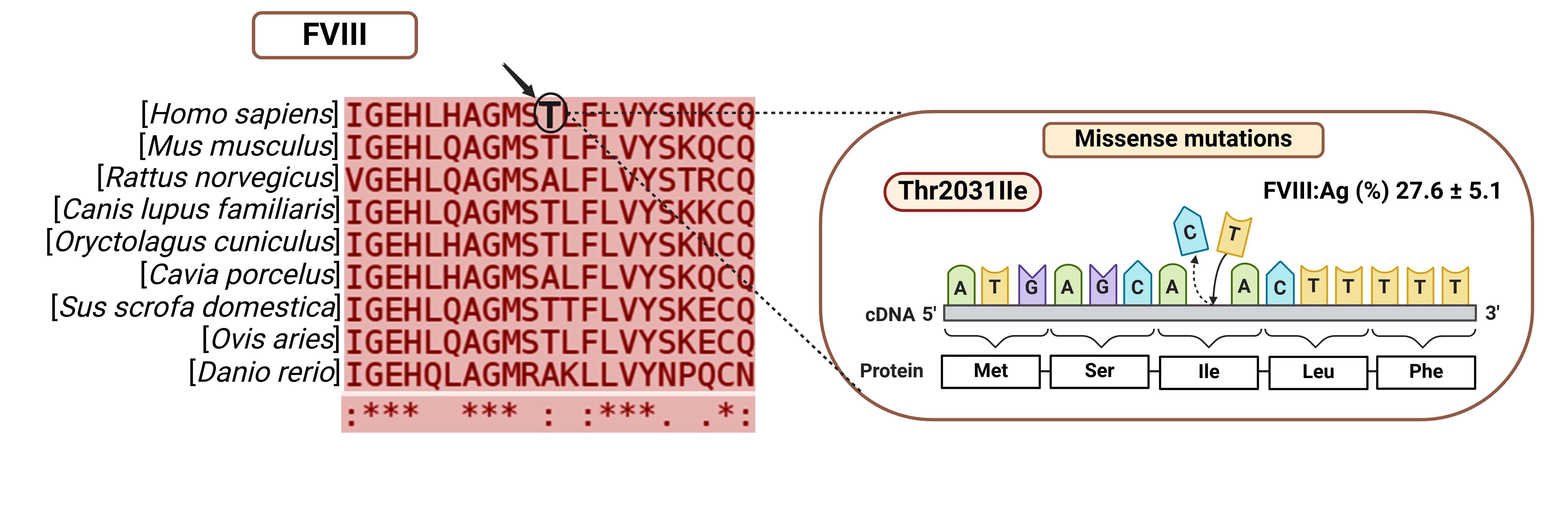

Supplement: S2 Fig — Multiple alignment of FVIII sequences, corresponding to Thr1898 homologous region in Homo sapiens, Mus musculus, Rattus norvegicus, Canis lupus familiaris, Oryctolagus cuniculus, Cavia porcelus, Sus scrofa domestica, Ovis aries and Danio rerio. The mutated amino acid can be seen at the center; the homologous amino acid in mice corresponds to Thr2031 in FVIII (in black). Abbreviations: F, factor; A, Antigen. (TIFF) [file pone.0321864.s002.tiff]

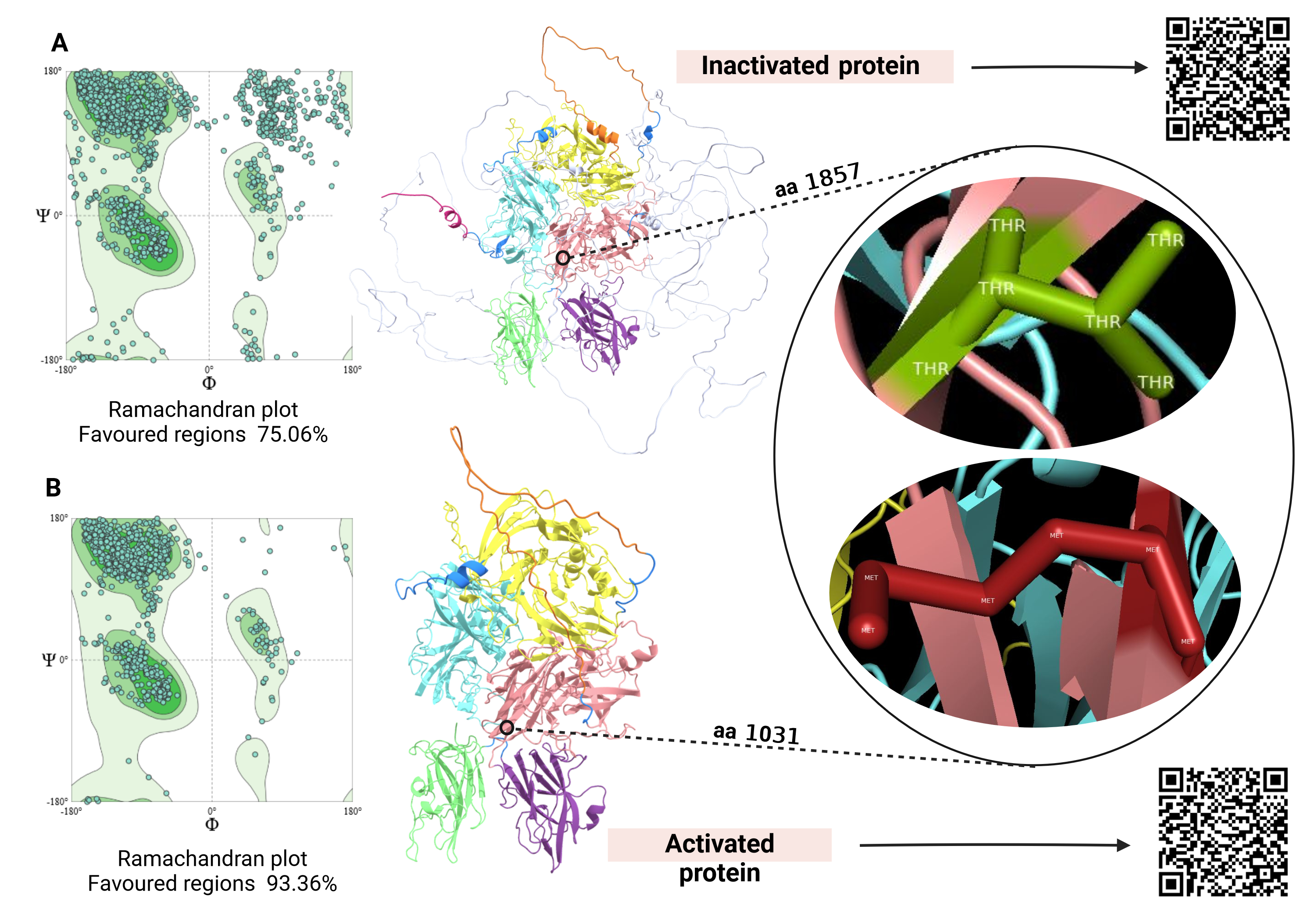

Supplement: S3 Fig — A) Graphic representation of the structure of inactive FV as predicted by the SWISS MODEL tool and the Ramachandran plot. B) Graphic representation of the structure of activated FV as predicted by the SWISS MODEL tool and the Ramachandran plot. Pymol visualization of WT threonine (green) and the mutated amino acid methionine (red). The QR code in the figure provides access to the 3D representation of each protein in the Sketchfab repository. Direct links: inactive FV, https://n9.cl/fvinactive; activated FV, https://n9.cl/fvactive_. (TIF) [file pone.0321864.s003.tif]

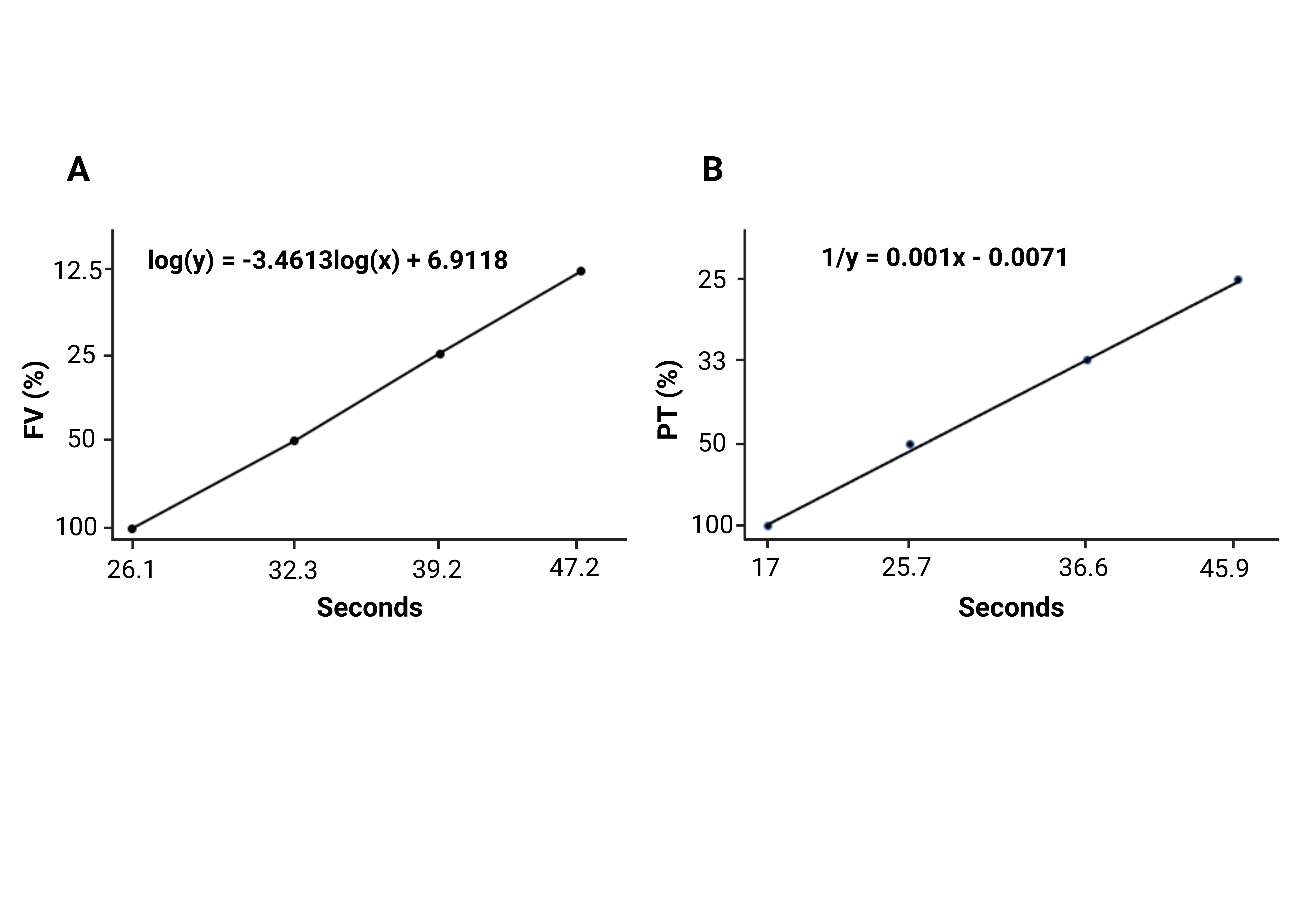

Supplement: S5 Fig — A) Calibration curve for FV. B) Calibration curve for PT. Abbreviations: FV, factor V; PT, prothrombin time. (TIF) [file pone.0321864.s005.tif]
