# Supplementary material for: Development of a novel and viable knock-in factor V deficiency murine model: Utility for an ultra-rare disease
Source: PLoS One. 2025 Jun 2;20(6):e0321864. doi: 10.1371/journal.pone.0321864 (PMC12129228; doi:10.1371/journal.pone.0321864)
Supplement: S4 Table — The WT allele is shown in the first raw for comparison. Edited alleles can be formed by base substitutions (marked in green letters), base deletions (marked by -) or base insertions (marked in red). The sought after allele is highlighted in blue). (DOCX) [file pone.0321864.s004.docx]

**S4 Table. Sequences of the alleles found in edited pups.** The WT allele is shown in the first raw for comparison. Edited alleles can be formed by base substitutions (marked in green letters), base deletions (marked by -) or base insertions (marked in red). The sought after allele is highlighted in blue).

| **Pup ID** | **ORF change** | **Sequence of the allele** | **% Reads** |
| --- | --- | --- | --- |
| **WT sequence** |  | TTGGAGAAAACCAGGTAGCTGGCATGCAAACGCCATTT |  |
|  |  |  |  |
| **Edited #1 (founder)** | -4 | TTGGAGAAAACC----AGCTGGCATGCAAACGCCATTT | 42.3 |
|  | 0 | TTGGAGAAAA**T**CAGGTAGCTGGCATGCAAA**T**GTTT | 47.6 |
|  |  |  |  |
| **Edited #2** | -9 | TTGGAGAAAA---------TGGCATGCAAACGCCATTT | 25.1 |
|  | -20 | TTGGAGA--------------------AAACGCCATTT | 31.1 |
|  | -11 | TT-----------GGTAGCTGGCATGCAAACGCCATTT | 43.8 |
|  |  |  |  |
| **Edited #3** | -7 | TTGGAGAAAACCAGG-------CATGCAAACGCCATTT | 100 |
|  |  |  |  |
| **Edited #4** | -7 | TTGGAGAAAACCAGGT-------ATGCAAACGCCATTT | 45.6 |
|  | 1 | TTGGAGAAAACCAGGTA**A**GCTGGCATGCAAACGCCATTT | 26.9 |
|  | 0 | WT | 27.4 |
|  |  |  |  |
| **Edited #5** | 0 | TTGGAGAAAA**T**CAGGTAGCTGGCATGCAAACGCCATTT | 100 |
|  |  |  |  |
| **Edited #6** | -20 | TTGGAGAAAACCAGG--------------------TTT | 24.4 |
|  | -20 | TTGGAGA--------------------AAACGCCATTT | 15.6 |
|  | 0 | TTGGAGAAAA**T**CAGGTAGCTGGCATGCAAACGCCATTT | 23.6 |
|  | -7 | TTGGAGAAAACCA-------GGCATGCAAACGCCATTT | 32.9 |
|  |  |  |  |
| **Edited #7** | -11 | TTGGAGAAAACCAGGTA-----------AACGCCATTT | 25.1 |
|  | 1 | TTGGAGAAAACCAGGTA**A**GCTGGCATGCAAACGCCATTT | 50.2 |
|  | 0 | WT | 24.7 |
|  |  |  |  |
| **Edited #8** | 0 | TTGGAGAAAA**T**CAGGTAGCTGGCATGCAAACGCCATTT | 100 |
|  |  |  |  |
| **Edited #9** | 0 | TTGGAGAAAA**T**CAGGTAGCTGGCATGCAAACGCCATTT | 49 |
|  | -4 | TTGGAGAAAACC----AGCTGGCATGCAAACGCCATTT | 51 |
|  |  |  |  |
| **Edited #10** | 0 | TTGGAGAAAA**T**CAGGTAGCTGGCATGCAAACGCCATTT | 100 |
|  |  |  |  |
| **Edited #11** | 0 | TTGGAGAAAA**T**CAGGTAGCTGGCATGCAAACGCCATTT | 52.8 |
|  | -3 | TTGGAGAAAACCAGGT---TGGCATGCAAACGCCATTT | 47.2 |
|  |  |  |  |
| **Edited #12** | -21 | TAGACAC---------------------AGCTGGCATGCAAACGCCATTT | 100 |
