# Supplementary material for: Development of a novel and viable knock-in factor V deficiency murine model: Utility for an ultra-rare disease
Source: PLoS One. 2025 Jun 2;20(6):e0321864. doi: 10.1371/journal.pone.0321864 (PMC12129228; doi:10.1371/journal.pone.0321864)
Supplement: S6 Table — Factor V measurements (expressed as % and seconds), prothrombin time measurements (expressed as %, seconds, and INR), and activated partial thromboplastin time measurements (expressed in seconds) for the WT, HZ (heterozygous), and HM (homozygous) groups. aPercentage obtained from the standard curve based on the plasma WT pool. bINR calculated as the ratio of the individual sample value to the normal sample value raised to the ISI. (DOCX) [file pone.0321864.s006.docx]

**S6 Table. Coagulometric measurements**. Factor V measurements (expressed as % and seconds), prothrombin time measurements (expressed as %, seconds, and INR), and activated partial thromboplastin time measurements (expressed in seconds) for the WT, HZ (heterozygous), and HM (homozygous) groups.

| **Group** | **Sex** | **FV (%)^a^** | **FV (sec)** | **PT (%)^a^** | **PT (sec)** | **INR^b^** | **APPT (sec)** |
| --- | --- | --- | --- | --- | --- | --- | --- |
| WT | Female | 74.20 | 30.0 | 82.64 | 19.2 | 1.1586 | 36.6 |
| WT | Female | 109.04 | 26.8 | 94.34 | 17.7 | 1.0500 | 35.4 |
| WT | Female | 168.29 | 23.6 | 94.34 | 17.7 | 1.0500 | 34.7 |
| WT | Male | 165.88 | 22.7 | 107.53 | 16.4 | 0.9575 | 31.4 |
| WT | Male | 92.76 | 28.1 | 106.38 | 16.5 | 0.9645 | 33.9 |
| WT | Male | 129.20 | 25.5 | 92.59 | 17.9 | 1.0644 | 33.8 |
| HZ | Female | 80.93 | 27.9 | 84.75 | 18.9 | 1.1368 | 40.9 |
| HZ | Female | 84.02 | 27.6 | 80.00 | 19.6 | 1.1879 | 37.1 |
| HZ | Female | 93.02 | 26.8 | 86.21 | 18.7 | 1.1222 | 37.1 |
| HZ | Male | 155.57 | 23.1 | 89.29 | 18.3 | 1.0933 | 38.0 |
| HZ | Male | 110.49 | 25.5 | 90.09 | 18.2 | 1.0860 | 36.5 |
| HZ | Male | 59.46 | 30.5 | 86.21 | 18.7 | 1.1222 | 33.9 |
| HM | Female | 26.08 | 38.7 | 58.14 | 24.3 | 1.5408 | 51.4 |
| HM | Female | 15.47 | 45.0 | 53.19 | 25.9 | 1.6644 | 48.7 |
| HM | Female | 22.09 | 40.6 | 64.52 | 22.6 | 1.4113 | 48.7 |
| HM | Male | 30.18 | 37.1 | 62.50 | 23.1 | 1.4492 | 45.7 |
| HM | Male | 26.55 | 38.5 | 71.94 | 21.0 | 1.2913 | 43.0 |
| HM | Male | 26.55 | 38.5 | 60.61 | 23.6 | 1.4872 | 43.9 |

^a^ Percentage obtained from the standard curve based on the plasma WT *pool*.

^b^ INR calculated as the ratio of the individual sample value to the normal sample value raised to the ISI.
